# Internal Cylinder Identification Based on Different Transmission of Longitudinal and Shear Ultrasonic Waves

**DOI:** 10.3390/s21030723

**Published:** 2021-01-21

**Authors:** Wen-Bei Liu, Wen-Bo Yan, Huan Liu, Cheng-Guo Tong, Ya-Xian Fan, Zhi-Yong Tao

**Affiliations:** 1 Guangxi Key Laboratory of Wireless Wideband Communication and Signal Processing, Guilin University of Electronic Technology, Guilin 541004, China; lwb2019@guet.edu.cn (W.-B.L.); tcg@guet.edu.cn (C.-G.T.); yxfan@guet.edu.cn (Y.-X.F.); 2 Key Lab of In-Fiber Integrated Optics, Ministry Education of China, Harbin Engineering University, Harbin 150001, China; yanwenbo83@163.com (W.-B.Y.); liuhuana@hrbeu.edu.cn (H.L.); 3 Academy of Marine Information Technology, Guilin University of Electronic Technology, Beihai 536000, China

**Keywords:** elastic waves, laser ultrasound, Fizeau fiber interferometer, defect identification

## Abstract

We have built a Fizeau fiber interferometer to investigate the internal cylindrical defects in an aluminum plate based on laser ultrasonic techniques. The ultrasound is excited in the plate by a Q-switched Nd:YAG laser. When the ultrasonic waves interact with the internal defects, the transmitted amplitudes of longitudinal and shear waves are different. The experimental results show that the difference in transmission amplitudes can be attributed to the high frequency damping of internal cylinders. When the scanning point is close to the internal defect, the longitudinal waves attenuate significantly in the whole defect area, and their amplitude is always smaller than that of shear waves. By comparing the transmitted amplitudes of longitudinal and shear waves at different scanning points, we can achieve a C scan image of the sample to realize the visual inspection of internal defects. Our system exhibits outstanding performance in detecting internal cylinders, which could be used not only in evaluating structure cracks but also in exploring ultrasonic transmission characteristics.

## 1. Introduction

In the aeronautics and automotive industry, there is a high demand for the quality of mechanical structures and materials. The cavities, grooves, and other types of defects generated during processing can affect the performance of the equipment and cause potential safety hazards. Therefore, harmful defects should be detected and be properly treated in time. In order to achieve safe and efficient detection for different kinds of manufacturing damages, the non-destructive evaluation (NDE) technology has been rapidly developed in various fields [1,2,3,4,5,6,7]. In NDEs, the detection of surface cracks has been paid much attention. For instance, Hong et al. used piezoelectric ultrasonic transducers to detect defects in plastic pipes [8], whereas Edwards et al. studied the relationship between depth of defects and ultrasonic signals by using the electro-magnetic acoustic transducers [9]. Campman et al. imaged surface defects in aluminum plates with laser ultrasonic methods [10] and developed the related methods and theories of wave propagation in marine seismic waves for applications [11,12]. In these works, researchers focused on surface defects and usually analyzed the reflected or transmitted ultrasound to obtain information about the cracks. Compared to surface defects, internal defects cannot be observed visually. In addition, influenced by the internal defects, the ultrasonic waves would experience amplitude attenuation, reflection, and scattering, which makes the detected signals too complex to be analyzed [13,14]. Therefore, the way to effectively detect and evaluate the internal defects is of great significance.

Due to the limitation of sensor size, the traditional piezoelectric transducer has limited spatial resolution, which makes it difficult to work normally in harsh environments such as high temperature, high radioactivity, and complex surfaces. With the development of NDEs, laser ultrasonic technology, as an effective method to solve these problems, has been widely used for years [15,16,17,18,19,20]. According to different detection requirements, laser ultrasonic technology can excite ultrasound through the thermo-elastic mechanism or ablation mechanism, which has the advantages of non-contact, high spatial resolution, and remote diagnosis. Based on a finite element method, Dai et al. obtained surface displacements of a 20 mm thick aluminum plate with various surface notch orientations [21] while Guan et al. modeled a surface notch with a depth of 200 μm, which is smaller than the center wavelength of Rayleigh waves [22]. Sohn et al. used the scanning laser-line source technique to investigate the interaction of surface-breaking flaws with ultrasonic waves [23]. Li et al. analyzed the ultrasonic frequency characteristics of a partially closed surface crack [24]. Zhou et al. numerically and experimentally investigated the reflection and transmission properties of a surface notch on an aluminum plate [25]. Wang et al. measured surface slot width using laser-generated Rayleigh waves [26]. Harb et al. proposed a fast imaging method for metal plate damage using zero-lag cross-correlation imaging conditions in the frequency domain [27]. In the above studies, ultrasonic signals were detected by spatial light paths system, laser interferometer, laser vibration meter, or other devices. Compared with these detection devices, the optical fiber interferometer not only maintains the advantages of high spatial resolution and high sensitivity but also overcomes the disadvantages of cumbersome and difficult adjustment of the spatial optical path, which can adapt to various detection conditions with high stability [28,29,30]. Moreover, the fiber interferometer can be built simply by using several kinds of optical fiber devices. The convenience of the system allows users to quickly master the operation method, which has good application potential in the field of NDE, especially for internal defects.

In this paper, we build a Fizeau fiber interferometer system to detect the size and shape of internal cylindrical defects. By using a precisely motorized positioning system, we obtain the ultrasonic signals at different positions within a two-dimensional area of the sample. We have found that the amplitudes of ultrasonic waves can be evidently affected by the internal defects and that the longitudinal wave attenuation is much larger than that of shear waves. Based on the relative intensity of the transmitted shear and longitudinal elastic waves, the shapes of internal cylinders have been imaged by carefully scanning the two-dimensional areas. In the following section, we build the proposed Fizeau fiber interferometer and introduce the experimental equipment. The waveform signals of transmitted ultrasonic waves are analyzed to illustrate the different attenuation effects of the internal defects on the longitudinal and shear waves in Section 3. In Section 4, by observing the trend of the shear wave amplitudes, we propose a data processing method to achieve the C scan images of the internal defects. Then, we spatially estimate the location, size, and shape of the prefabricated internal cylinders. Finally, the major results on internal defect identification based on the proposed Fizeau fiber interferometer and data processing method are summarized in Section 5.

## 2. Laser Ultrasonic Detection

### 2.1. Fizeau Fiber Interferometer

**Figure 1:**
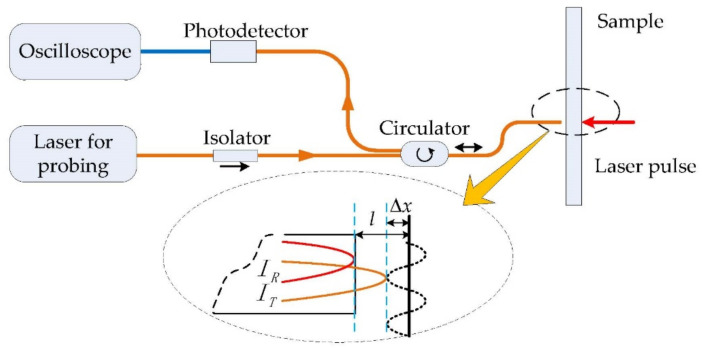
Schematic diagram of Fizeau fiber interferometer and its working principle.

The Fizeau fiber interferometer [31] was designed to study the ultrasonic field on the relevant surface of the sample with internal defects. The schematic diagram of the interferometer is shown in Figure 1. The structure of Fizeau fiber interferometer is composed of single-mode fibers used to transmit signal light and reference light. The narrow linewidth tunable laser (TLT-1500 LaseGen, CA, USA) with output wavelength of 1550 nm and power of 15 mW is used as the detection light source of the interferometer. The continuous detection light is transmitted to the circulator through the isolator. Here, the isolator is used to prevent the detection light returning to the laser to ensure the stability of the interferometer.

When the detection laser outputs from the circulator, 4% of the energy at the end of the fiber would be reflected as the reference light. Some of the transmitted light would radiate on the surface of the sample and then be reflected by the surface of the sample. Most of the light would enter the fiber again. The two beams satisfy the coherent condition, and the vibration on the surface of the sample can change the optical path difference of the two beams. The coherent light intensity satisfies the following formula:

(1) $$I=I_{R}+I_{T}+2\sqrt{I_{R}I_{T}}\cos\Delta \varphi$$

(2) $$\Delta \varphi =\frac{4\pi n_{0}\left(l+\Delta x\right)}{\lambda }$$

where *I* is the interference light intensity, Δ*φ* is the phase difference, Δ*x* is the vibration displacement, and $n_{0}$ is the air refractive index. By adjusting the length of the interference cavity *l*, we can make $4n_{0}l=(2k+0.5)\lambda$, where *k* is an integer. Then, the coherent light intensity has a linear relationship with the very small vibration displacement. The intensity of interference light is converted into electrical signal by the photoelectric detector and displayed on the oscilloscope. We can thus get the ultrasonic signals detected by the Fizeau fiber interferometer.

### 2.2. Experimental Processes

**Figure 2:**
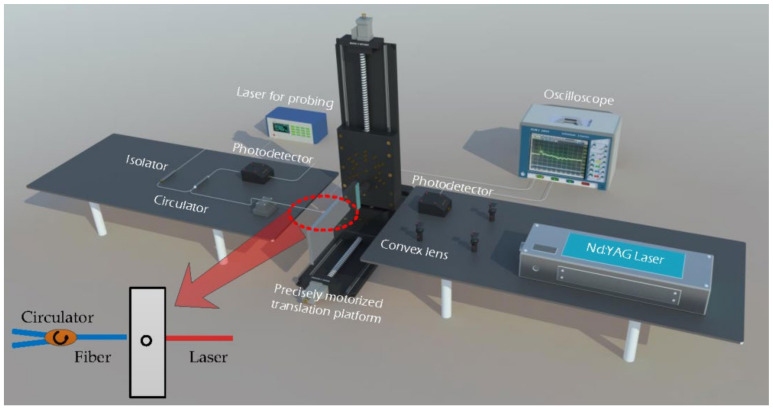
Schematic diagram of experimental setup. The inset also shows the relative position of the fiber and the pulse laser on the sample.

The experimental setup is schematically shown in Figure 2. In the experiments, a pulsed laser with a wavelength of 1064 nm and a duration of 6 ns was generated by an Nd:YAG laser (DAWA-200 from Beamtech Optronics Co., Ltd., Beijing, China), and the repetition rate of the laser source was selected as 3 Hz. The average laser pulse energy was controlled around 20 mJ to guarantee that the ultrasonic wave was generated. The splitter lens was used to divide the pulse laser into two beams. One beam of the laser was focused into a spotlight through a focus lens with a 100 mm focal length to excite the ultrasonic waves, and the other beam was received by a space photodetector (KG-PR-1G-A-PC from Beijing Conquer Optics Science & Technology Co., Ltd., Beijing, China) as the trigger signal to the digital oscilloscope (MOS-S-204A from Keysight Technologies, Inc., Santa Rosa, CA, USA). The position of the probe fiber and the pulsed laser relative to the sample is also depicted in the inset of Figure 2.

**Figure 3:**
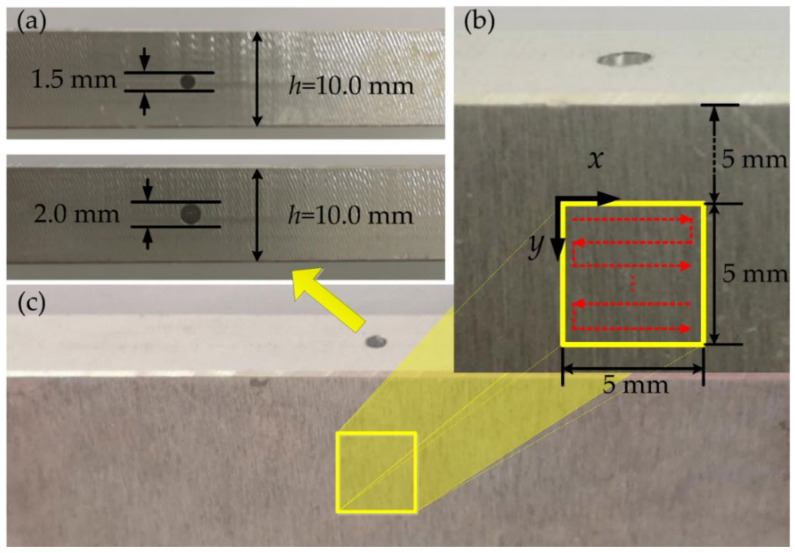
Aluminum plate samples with the different holes on one side. (**a**) Two holes with 1.5 mm and 2.0 mm diameters. (**b**) Scanning area and path. (**c**) Aluminum plate with the hole and scanning area.

The two aluminum plates with length of 300 mm, width of 100 mm, and thickness of 10 mm were selected and held alternately by a two-dimensional precisely motorized translation stage (Beijing Optical Century Instrument Co., Ltd., Beijing, China) as shown in Figure 2. The two aluminum plate samples with the thickness *h* = 10 mm were prepared for the test as shown in Figure 3a. We drilled a 2.0 mm diameter hole vertically on the upper side of one aluminum plate, while on the other aluminum plate, we used a 1.5 mm diameter drill bit to create a vertical hole, and then reprocessed it with a 1 mm diameter drill at the same location to create the hole with the diameter varying from 1.5 mm to 1 mm. The precise electric translation table could move in a two-dimensional direction, and the repetition position accuracy was less than 5 μm. Before the test, we adjusted the relative position of the sample and the optical fiber probe so that the bottom of the internal defect was located in the center of the scanning area. As shown in Figure 3b, the scanning area with the size of 5 mm × 5 mm was 5 mm lower than the upper edge of the aluminum plate, the scanning step size was 0.2 mm, and the red arrows denote the scanning direction. Then, the probe laser was focused on the sample surface with a fiber probe. During the scanning process, we kept the position of the optical fiber probe and laser focus unchanged and only used the translation stage to move the samples. This scanning method can avoid the additional phase change and laser energy fluctuation caused by the movement of the fiber probe, which effectively improves the stability and efficiency of the system.

## 3. Transmission of Longitudinal and Shear Waves

**Figure 4:**
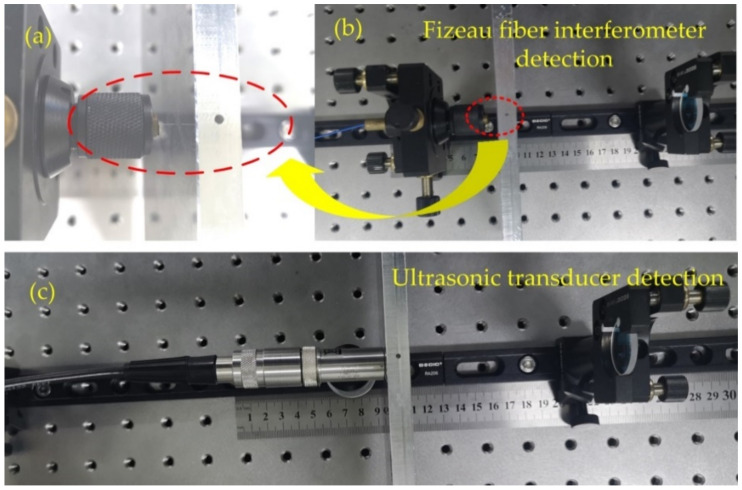
Fiber end face of the Fizeau fiber interferometer (**a**), and the interferometer (**b**) and ultrasonic transducer (**c**) detection.

To illustrate the advantages of the proposed fiber interferometer, we compared our interferometer with the traditional ultrasonic devices. The fiber end face of the Fizeau fiber interferometer is presented in Figure 4a, which is the detailed enlarged view of the fiber probe in the actual picture of the detection as shown in Figure 4b. Figure 4c shows the detection by using an ultrasonic transducer with a center frequency of 1 MHz. The ultrasonic transceiver model of the ultrasonic probe is Olympus 5077PR.

**Figure 5:**
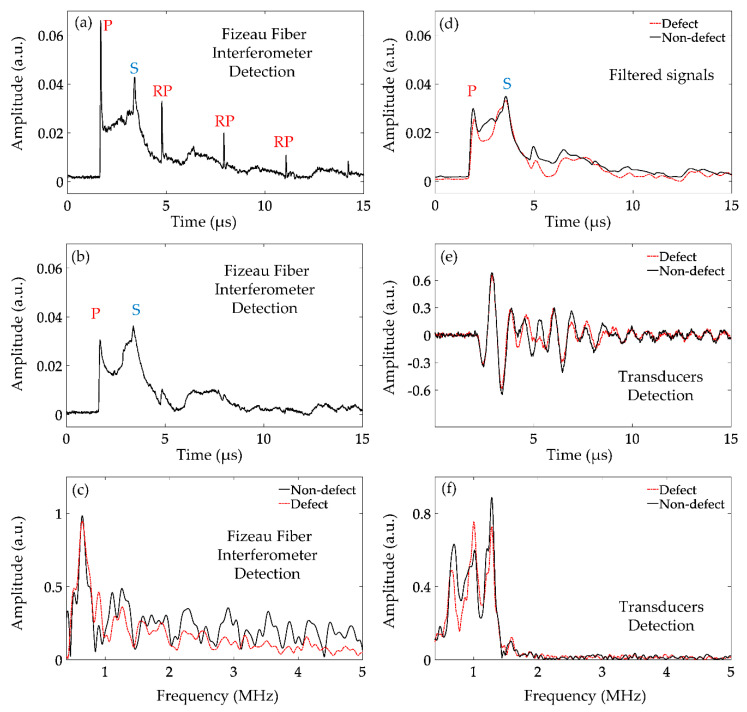
Detected time and frequency domain ultrasonic signals: Fizeau fiber interferometer signals without (**a**) and with (**b**) defects, as well as their frequency spectra (**c**) and the low-pass filtered signals (**d**). Signals (**e**) and their frequency spectra (**f**) detected by the ultrasonic transducers.

The typical ultrasonic time and frequency domain signals obtained by the proposed interferometer are shown in Figure 5a–c. The detected ultrasonic signal without defects is shown in Figure 5a. By analyzing the signal, we can find that the Fizeau fiber interferometer can detect the two ultrasonic modes: longitudinal and shear waves. A longitudinal wave is a kind of compression wave whose vibration direction is parallel to the propagation direction. The interference optical path difference caused by its vibration will be the largest, so the amplitude change of longitudinal wave signal detected by Fizeau fiber interferometer is the strongest. The thickness of the aluminum plate is 10 mm. The propagation velocity of longitudinal waves in an aluminum plate is about 6400 m/s [32]. According to the velocity formula, the time of receiving longitudinal waves by Fizeau fiber interferometer can be calculated by

(3) $$t_{P}=\frac{v_{P}}{h},$$

where *t_p_* and *v_p_* are the receiving time and the propagation velocity of longitudinal waves, respectively. The results show that the peak at *t_p_* = 1.56 μs was the longitudinal wave signal (denoted by P). In addition, due to the influence of the sample boundaries, the reflected signals of longitudinal waves (denoted by RP) were also detected. The propagation velocity of shear waves in the aluminum plate was about 3150 m/s [32]. According to the following velocity formula, the shear wave receiving time can be calculated.

(4) $$t_{S}=\frac{v_{S}}{h},$$

where *t_s_* and vs. are the receiving time and the propagation velocity of shear waves, respectively. It was calculated that the lower peak at *t_s_* = 3.21 μs is the shear wave signal (denoted by S).

When defects are detected by the optical fiber interferometer, the obtained ultrasonic signals were different as shown in Figure 5b. Compared with the defect-free results, we find that the amplitudes of P and S waves are both attenuated. However, it can be also observed that the attenuation degrees of these two kinds of ultrasonic signals are different. The amplitude attenuation of the P wave is more severe. The frequency domain expansions of ultrasonic signals measured with and without defects are depicted in Figure 5c by the red dashed and black solid lines, respectively. We find that the high-frequency components of the signal without defects are much larger. As shown in Figure 5a,b, the P wave attenuation is severe. We suppose that the P wave contains more frequency components higher than 2 MHz, because when defects are detected, they are severely attenuated in the time and frequency domain, respectively. In order to verify this point of view, we chose a Butterworth low-pass filter with a cutoff frequency of 2 MHz to the ultrasonic signals measured with and without defects as shown in Figure 5d. The black solid line represents the signal processed by filtering when there is no defect. It can be found that both P wave and RP waves are filtered out. This proves that P waves contain more high-frequency components than S waves. The red dashed line indicates the signal processed by filtering when there are defects. It can be found that the signal becomes smooth but does not change dramatically from the nonfiltered signal. This not only proves that P waves contain more high-frequency components but also confirms that the attenuation of P waves is more severe than that of S waves when the ultrasound passes through defects. The internal defect does affect the ultrasonic wave transmission, and the effects on the P and S waves are quite different. This is because the angle of the longitudinal and transverse wave propagation is different when the ultrasound is excited by lasers [32]. The propagation direction of P waves is always perpendicular to the aluminum plate surface, whereas the S wave propagates in a certain angle; thus it can bypass the defect. Therefore, it seems very promising that we can identify the internal defect by carefully testing the different transmissions of P and S waves.

At the same time, we also used the ultrasonic transducers with the center frequency of 1 MHz to detect the samples with and without defects as shown in Figure 5e by the red dashed and black solid lines, respectively, as well as their frequency spectra in Figure 5f. It is difficult to directly observe the difference between the two ultrasound signals received by the transducer due to its much larger size. In comparison, the proposed optical fiber interferometer has more advantages in the detection of small internal defects.

**Figure 6:**
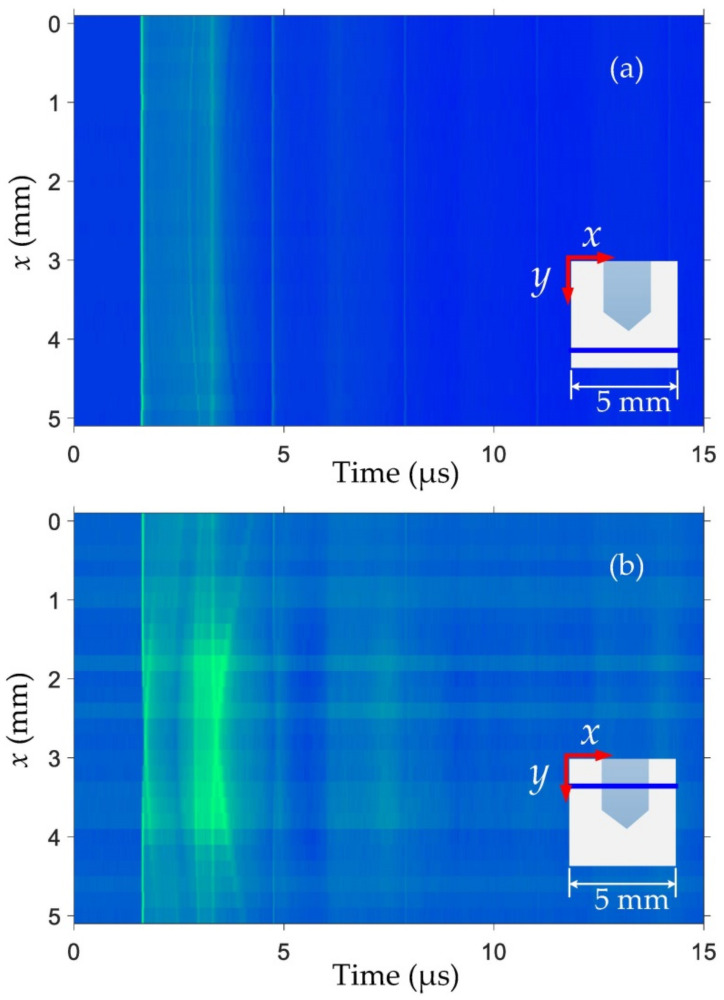
B scan image along the horizontal direction in the non-defect (**a**) and defective (**b**) regions. The scanning route is also depicted by the bold blue line in each insect. To compare the attenuation of P waves and S waves, the signal amplitudes are both normalized by their own P wave intensities.

In order to further investigate the effects of internal defects on the transmission of ultrasonic waves, we selected the same horizontal scanning in the non-defective and defective regions and obtained the B scan images along the *x*-axis by data processing, shown respectively as Figure 6a,b. We have normalized the ultrasonic waveform by the P wave intensity without defects as the standard to facilitate the observation of the amplitude changes of ultrasonic waves. The intensity of the amplitude is represented by the color from blue to green: the greener the color, the greater the intensity of the signal. Referring to the ultrasonic mode analysis above, we can clearly observe the P wave and the S wave, and a series of boundary reflected waves in Figure 6a, when the scanning does not encounter any defects. Moreover, it has been proved that the amplitude intensity of recorded ultrasonic signals is very stable in the scanning process. In contrast, the ultrasonic waveform would change significantly when the interferometer was scanning in the defective region shown in Figure 6b. It can be seen that the amplitude of P waves will decrease as the scanning position gradually approaches the internal defect, while the amplitude of S waves increases due to the high-frequency damping phenomenon. Therefore, we have observed a bright region appearing around 3.3 μs as the scanning position moves to the location of the internal defect. As the scanning point moves out of the defective region, the ultrasonic waveform is restored to a non-defective state and remains stable again. In addition, we also found the arc-shaped ultrasonic from the diagram due to the cylindrical shape of the predrilled internal defect, indicating the possibility of defect shape identification based on the recorded ultrasonic signals by the interferometer.

## 4. Visualization of Internal Cylinders

Based on the B scan results mentioned above, the existence of internal defects can be determined by the interferometer received ultrasonic waves. However, since the amplitude change of ultrasonic waves is a gradual process, the boundary of the defect cannot be accurately determined yet. Therefore, it is also necessary to investigate the C scan images to inspect the internal defects more intuitively. We found that the amplitudes of P and S waves have different degrees of attenuation due to the influence of internal defects, and the attenuation of P waves is more significant. Near the defect boundary, the amplitude relationship between the two types of ultrasonic waves changes, and the S wave amplitude will gradually approach and exceed that of the P waves. In order to determine the defect location, the maximum value of P wave amplitude and S wave amplitude are selected as *P*_max_ and *S*_max_, respectively. We set the parameter *K*, which is given by the following formula.

(5) $$K=\frac{S_{\max}}{P_{\max}}.$$

**Figure 7:**
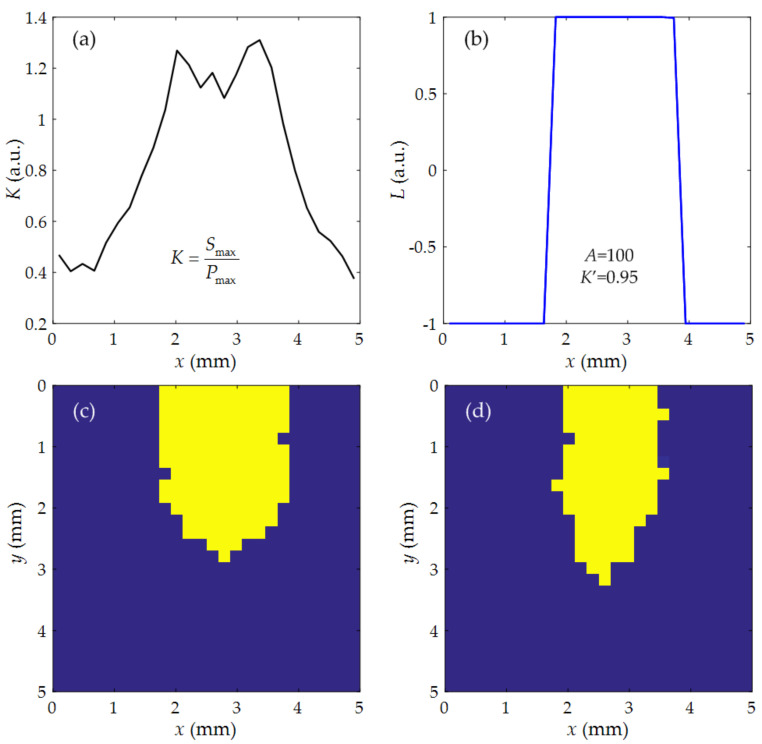
*K* (**a**) and *L* (**b**) parameters for the scanning signals along the *x*-axis and the C scan images of two samples with 2 mm (**c**) and 1.5 mm (**d**) diameter cylinders, where the yellow is for *L* = 1 and the blue is for *L* = −1.

The defect can change the value of *K* by attenuating P waves. Therefore, when scanning the area shown in Figure 3b, the value of *K* can be used to determine whether there are defects in the scanning position. A scanning track in the *x*-axis direction is selected, and the relationship between *K* and *x*-axis displacement is shown in Figure 7a. It can be seen that when the detection position moves into the location of the defect, *K* begins to increase significantly. In order to determine the defect boundary more accurately, we introduce the following formula.

(6) $$L=\tanh\left[A(K-K^{\prime })\right]$$

where *A* and *K*′ are both the measurement parameters, which can be calibrated by a hole with standard sizes. The relationship between *L* and *x*-axis displacement is shown in Figure 7b, where *A* = 100 and *K*′ = 0.95 are selected.

With the measured data from a standard hole, we can optimize the measurement parameters *A* and *K*′. The value of *A* can affect the change degree of *L* at the boundary. Choosing *A* = 100 satisfies the change judgment of *L* at the boundary and *K*′ is the criterion of defect boundary. When we select *A* = 100 and *K*′ = 0.95, *L* = 1 inside the defect, while *L* = −1 outside. It can be seen from the results that the boundary of defects can be accurately determined. The final imaging results of the whole scanning area are shown in Figure 7c,d for the two samples with 2 mm and 1.5 mm diameters, respectively. The yellow is for *L* = 1 and the blue is for *L* = −1. Thus, we can figure out the internal cylinders according to the different colors. The experimental results show that the scanning image obtained by this method is basically consistent with the characteristics of cylindrical defects in the samples, even for the cylinder with varying diameters. In addition, there were still some spots at the edges of the defects in the C scan images due to the measuring errors. Because our scanning step is 0.2 mm, the edge of the defect image has an error within 0.2 mm. This error can be further reduced by decreasing the scanning step. This method has nothing to do with the propagating media of ultrasounds, so it can also be easily applied to other metals.

## 5. Conclusions

We built a Fizeau fiber interferometer to detect the internal defects in aluminum plates. This system has the advantages of simple structure, high sensitivity, and high spatial resolution. Through experimental and theoretical analysis, we studied the interaction between internal defects and ultrasonic waves. We observed that the variation of the S waves and P waves is affected by internal defects based on the B scan images with different *y*-axis positions, and the phenomenon of different attenuation degree of waveforms in different modes are explained by the damping of high frequencies. Finally, by comparing the S wave amplitude of different scanning points with the set measurement parameters *A* and *K*′, the C scan images of the predrilled cylinders were obtained to implement the visual inspection of internal defects. It is very impressive that the C scan images are consistent with the actual features of the internal defects based on the Fizeau fiber interferometer system, which provides a more simple way to measure the size, position, and shape of internal defects. The measurement error is dependent on the step of scanning, which can be decreased according to the resolution of translation stage.

In summary, the Fizeau fiber interferometer system can be effectively used to investigate the internal defects, and the measured results reveal the propagation characteristics of ultrasonic waves and their interaction mechanism with internal defects. The proposed system and methods not only enrich our knowledge of the interaction between defects and ultrasound, but also find a simple way to detect the structures in high resolution, which have good application prospects in the field, such as NDE, ultrasonic measurements, and biomedical imaging.

## Data Availability

The data presented in this study are available on request from the corresponding author.
